# Die neue Parkinson-Schmerzklassifikation (PSK)

**DOI:** 10.1007/s00115-021-01258-y

**Published:** 2022-01-28

**Authors:** V. Mylius, S. Perez Lloret, C. S. Brook, M. T. Krüger, S. Hägele-Link, R. Gonzenbach, J. Kassubek, S. Bohlhalter, J. P. Lefaucheur, L. Timmermann, G. Kägi, F. Brugger, D. Ciampi de Andrade, J. C. Möller

**Affiliations:** 1 Klinik für Neurologie und Neurorehabilitation, Kliniken Valens, Valens, Schweiz; 2 Klinik für Neurologie, Philipps Universität Marburg, Marburg, Deutschland; 3 Klinik für Neurologie, Kantonsspital St. Gallen, St. Gallen, Schweiz; 4 Biomedical Research Center (CAECIHS-UAI), National Research Council (CONICET), Buenos Aires, Argentinien; 5 Klinik für Neurochirurgie, Kantonsspital St. Gallen, St. Gallen, Schweiz; 6 Klinik für Neurologie, Universität Ulm, Ulm, Deutschland; 7 Neurozentrum, Luzerner Kantonsspital and Universität Zürich, Luzern, Schweiz; 8 EA 4391, Unité de Neurophysiologie Clinique, Hôpital Henri Mondor, Assistance Publique – Hôpitaux de Paris, Faculté de Médecine de Créteil, Université Paris-Est-Créteil, Créteil, Frankreich; 9 Universitätsklinik für Neurologie, Inselspital, Universitätsspital Bern, Bern, Schweiz; 10 Pain Center, LIM-62, Departamento de Neurologia da Faculdade de Medicina da Universidade de Sao Paulo, Hospital das Clínicas, Sao Paulo, Brasilien; 11 Center for Neuroplasticity and Pain (CNAP), Department of Health Science and Technology, Faculty of Medicine, Aalborg University, Aalborg, Dänemark; 12 Parkinsonzentrum, Zentrum für Neurologische Rehabilitation, Zihlschlacht, Schweiz

**Keywords:** Schmerzen, Fragebogen, Noziplastisch, Neuropathisch, Nozizeptiv, Pain, Questionnaire, Nociplastic, Neuropathic, Nociceptive

## Abstract

**Hintergrund:**

Chronische Schmerzen sind ein häufiges nichtmotorisches Symptom beim Patienten mit M. Parkinson.

**Ziel der Arbeit:**

Da die Zuordnung von Schmerzen bei Parkinson-Patienten nicht einfach ist, haben wir eine neue Parkinson-Schmerzklassifikation (PSK) mit einem zugehörigen Fragebogen validiert und nun ins Deutsche übersetzt.

**Methoden:**

Zunächst kann der Zusammenhang von Schmerzen mit der Parkinson-Erkrankung anhand von vier Fragen festgestellt werden, bevor die weitere hierarchisch aufgebaute Unterteilung in eine von drei Schmerzkategorien erfolgt (neuropathisch, nozizeptiv und noziplastisch).

**Ergebnisse:**

Bei 77 % der Patienten (122/159) der initialen Validierungsstudie lagen Parkinson-assoziierte Schmerzen vor, wobei bei 87 (55 %) Patienten nozizeptive, bei 36 (22 %) noziplastische und bei 24 Patienten (16 %) neuropathische Schmerzen vorlagen. Die Studie zeigte eine hohe Validität des Schmerzfragebogens bei einer moderaten Intra- und Interraterreliabilität. Die deutsche Version des Fragebogens wurde nach Anpassungen bei 30 Patienten angewendet.

**Diskussion:**

Der PSK-Fragebogen ist ein valides und reliables Werkzeug, um Parkinson-assoziierten Schmerz von nicht-Parkinson-assoziiertem Schmerz zu unterscheiden und die Schmerzen einer Kategorie zuzuordnen, was die weitere Diagnostik und Therapie erleichtert.

**Zusatzmaterial online:**

Die Onlineversion dieses Beitrags (10.1007/s00115-021-01258-y) enthält weitere Infomaterialien. Beitrag und Zusatzmaterial stehen Ihnen auf www.springermedizin.de zur Verfügung. Bitte geben Sie dort den Beitragstitel in die Suche ein, das Zusatzmaterial finden Sie beim Beitrag unter „Ergänzende Inhalte“.

eAnhang 1. Der Parkinson-Schmerzklassifikations-Fragebogen (deutsche Übersetzung mit Erlaubnis nach [40]), eAnhang 2. Erläuterungen zum Parkinson-Schmerzklassifikations-Fragebogen (deutsche Übersetzung mit Erlaubnis nach [40])

## Abstract (en)

**Background:**

Chronic pain is a common non-motor symptom in patients with Parkinson’s disease (PD).

**Aim:**

To facilitate the diagnosis of pain in PD, we developed a new classification system the Parkinson’s disease pain classification system (PD-PCS) and translated the corresponding validated questionnaire into German.

**Methods:**

A causal relationship of the respective pain syndrome with PD can be determined by four questions before assigning it hierarchically into one of three pain categories (neuropathic, nociceptive and nociplastic).

**Results:**

In the initial validation study 77% of the patients (122/159) had PD-associated pain comprising 87 (55%) with nociceptive, 36 (22%) with nociplastic and 24 (16%) with neuropathic pain. The study revealed a high validity of the questionnaire and a moderate intrarater and interrater reliability. The questionnaire has been adapted into German and employed in 30 patients.

**Discussion:**

The PD-PCS questionnaire is a valid and reliable tool to determine the relationship of a pain syndrome with PD before classifying it according to the underlying category, facilitating further diagnostics and treatment.

## Hintergrund

Chronische Schmerzen sind ein häufiges Symptom bei M. Parkinson. Da die Zuordnung zur Parkinson-Erkrankung oft nicht einfach ist, haben wir eine neue Parkinson-Schmerzklassifikation (PSK) entworfen und validiert. Sie erlaubt die Unterscheidung von Parkinson-abhängigen und Parkinson-unabhängigen Schmerzen, bevor eine weitere Unterteilung anhand der Kriterien der International Association for the Study of Pain (IASP) erfolgt. Den bekannten Kategorien der neuropathischen und nozizeptiven Schmerzen wurde mit dem noziplastischen Schmerz ein neu beschriebener Mechanismus hinzugefügt. Der Fragebogen der englischen Originalversion wurde nun ins Deutsche übersetzt und bei 30 Patienten angewendet (s. Zusatzmaterial online).

Chronische Schmerzen (Dauer > 3 Monate) sind ein häufiges nichtmotorisches Symptom bei Patienten mit M. Parkinson [46]. Die Häufigkeit Parkinson-assoziierter chronischer Schmerzen nimmt mit der Erkrankungsdauer zu und wird zu Beginn der Erkrankung in der frühen motorischen Phase auf 20 % (typischerweise Schulter-Arm-Schmerzen) und in späteren Stadien auf 80 % geschätzt [3, 19, 32]. Schmerz gehört zu den Symptomen, die die Lebensqualität von Patienten mit Parkinson je nach Stadium und Begleitsymptomen beeinträchtigen [33, 35]. Insbesondere stärkere Schmerzen und Schmerzen im frühen bis mittleren Stadium der Erkrankung haben einen negativen Einfluss auf die Lebensqualität [40, 47].

Schmerzen sind oft direkt mit der Parkinson-Erkrankung assoziiert, was mit einer erhöhten Schmerzwahrnehmung durch Veränderungen der Schmerzverarbeitung erklärt werden kann. Die neuroanatomische Basis sind eine vermehrte Aktivierung schmerzverarbeitender kortikaler Strukturen bei geringer dopaminerger Stimulation sowie eine reduzierte deszendierende Schmerzhemmung [7, 27, 36, 38]. Zuletzt wurde ein Parkinson-Schmerztyp als eine nichtmotorische Unterform des M. Parkinson beschrieben [45].

In der klinischen Praxis ist die Unterscheidung Parkinson-abhängiger von Parkinson-unabhängigen Schmerzen oft nicht einfach, da Schmerzen im Alter ebenfalls häufig sind [18]. Daher haben wir eine Parkinson-Schmerzklassifikation (PSK) entworfen, die zunächst Parkinson-abhängige von Parkinson-unabhängigen Schmerzen differenziert, bevor eine mechanismenbasierte Unterteilung erfolgt. Dafür wurden, wie in der Schmerztherapie üblich, die bisher beim M. Parkinson beschriebenen Schmerzformen einem Schmerzmechanismus zugeordnet (neuropathisch, nozizeptiv und noziplastisch; [14]). Eine internationale Validierungsstudie konnte eine moderate Reliabilität und eine hohe Validität der Klassifikation zeigen [40]. Mit einem Fragebogen, der auch als Kurzversion online zur Verfügung steht, wird die Zuordnung erleichtert.

## Sind Schmerzen Parkinson-assoziiert?

**Figure 1:**
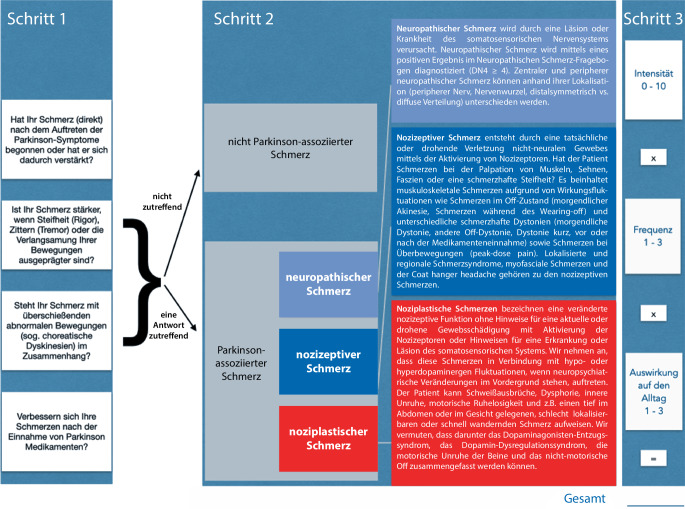

Erstes Ziel der Klassifikation ist es, Parkinson-assoziierte von nicht-Parkinson-assoziierten Schmerzen zu unterscheiden ([16, 37, 40]; Abb. 1). Die Klassifikation von Quinn und die Frage nach Parkinson-assoziierten Schmerzen aus dem Fragebogen für nichtmotorische Beschwerden bei M. Parkinson fließen hier in veränderter Form ein [11, 42]. Als Parkinson-assoziiert gelten Schmerzen, die früh mit den motorischen Symptomen auftreten, auf dopaminerge Medikation ansprechen oder durch die Parkinson-Erkrankung verstärkt werden. Zudem ist das Auftreten in der Off-Phase ein wichtiger Hinweis für Parkinson-assoziierte Schmerzen [11, 42]. Dazu gehören auch Schmerzen verbunden mit Dystonie (typisch: „early-morning off“). Seltener kann es auch bei Peak-dose-Dyskinesien zu Schmerzen kommen, wenn beispielsweise eine Arthrose vorliegt. Wenn mindestens eine der 4 Fragen mit „Ja“ beantwortet werden kann, ist eine Assoziation der Schmerzen mit der Parkinson-Erkrankung anzunehmen und es kann eine weitere Unterscheidung erfolgen. Anderenfalls liegt ein Parkinson-unabhängiger Schmerz vor und weitere Diagnostik wird erforderlich.

## Welche Schmerzkategorie liegt vor?

Bei Parkinson-Patienten können *neuropathische und nozizeptive Schmerzen* unterschieden werden [52]. Mit *noziplastischen Schmerzen* wird neuerdings eine weitere Kategorie auch für Schmerzen beim M. Parkinson definiert. Unser Fragebogen verwendet einen hierarchischen Algorithmus mit dem zunächst nach neuropathischen und nozizeptiven Schmerzen gefragt wird, bevor noziplastische Schmerzen klassifiziert werden.

*Neuropathische Schmerzen* werden als Schmerzen im Zusammenhang mit einer Erkrankung oder Läsion des somatosensorischen Systems definiert [5]. Neuropathischer Schmerz kann durch den typischen neuropathischen Charakter des Schmerzes diagnostiziert werden. Wir empfehlen die Anwendung des aus dem Französischen übertragenen Douleur-Neuropathique-Fragebogens (DN4; [6]). Wenn mindestens 4 von 10 Fragen mit „Ja“ beantwortet werden, liegt ein neuropathischer Schmerz vor. Bei Parkinson-Patienten kann peripherer neuropathischer Schmerz (z. B. radikulär) von zentral-neuropathischem Schmerz unterschieden werden (Lokalisation mit zentralem Muster, nicht peripher). Der DN4 ist eine einfache Möglichkeit, neuropathische Schmerzen festzustellen. Mit dem painDETECT liegt ein weiterer Fragebogen vor, der eine Unterscheidung zwischen neuropathischem und nozizeptivem Schmerz erlaubt und einen Graubereich mit möglicher neuropathischer Schmerzkomponente definiert [25].

Bei *nozizeptiven Schmerzen* liegt eine gegenwärtige oder drohende nichtneuronale Gewebsschädigung vor, die eine Nozizeptoraktivierung verursacht. Dabei kann die Palpation von Gelenk, Faszien, Sehnen oder Muskel schmerzhaft sein. Beim M. Parkinson beinhaltet dies muskuloskeletale Schmerzen durch motorische Fluktuationen wie Off-Phasen (morgendlicher Schmerz, „wearing-off“ und unvorhersehbare On-Off-Phänomene), die schmerzhafte Dystonie (morgendlicher Schmerz, die Off-Dystonie sowie die biphasischen Dyskinesien, die oft einen dystonen Charakter haben) und Schmerzen bei Peak-dose-Dyskinesien. Auch lokalisierte oder regionale Schmerzsyndrome wie das myofasziale Schmerzsyndrom [1, 26] und Nackenschmerzen bei orthostatischer Hypotonie („coat hanger headache“) werden hier aufgeführt. Diese Nackenschmerzen korrelieren mit dem Schweregrad der orthostatischen Hypotonie insbesondere bei der Multisystematrophie, können aber auch beim M. Parkinson vorkommen [4].

*Noziplastische Schmerzen* bezeichnen eine veränderte nozizeptive Funktion ohne Hinweise für eine aktuelle oder drohende Gewebsschädigung mit Aktivierung der Nozizeptoren oder Hinweisen für eine Erkrankung oder Läsion des somatosensorischen Systems (Ausschluss nozizeptiver und neuropathischer Schmerzformen; [22, 29, 31]). Beispielsweise können primären chronischen Schmerzsyndromen noziplastische Schmerzmechanismen zugrunde liegen (z. B. bei primär chronischem Kopfschmerz und dem komplex-regionalen Schmerzsyndrom; [49]). In vorherigen Publikationen wurden nichtneuropathische und nichtnozizeptive Schmerzen in der Regel unter „Verschiedene“ oder unter „psychomotorische Unruhe mit Schmerzen“ zusammengefasst [37, 52]. 2019 wurde die Bezeichnung noziplastischer Schmerz erstmals für zentrale Schmerzen und das Restless-legs-Syndrom beim M. Parkinson verwendet [34]. Bisher liegen, bis auf unsere Validierungsstudie [40], keine Arbeiten vor, die noziplastische Schmerzen beim M. Parkinson untersucht haben. Wir nehmen an, dass diese Schmerzen in Verbindung mit hypo- oder hyperdopaminergen Fluktuationen, wenn neuropsychiatrische Veränderungen im Vordergrund stehen, auftreten. Der Patient kann – gemäß unseres Fragebogens – Schweißausbrüche, Dysphorie, innere Unruhe, motorische Ruhelosigkeit und z. B. einen tief im Abdomen oder im Gesicht gelegenen, schlecht lokalisierbaren oder schnell wandernden Schmerz aufweisen. Wir haben darunter a priori das Dopaminagonistenentzugssyndrom, das dopaminerge Dysregulationssyndrom, die motorische Ruhelosigkeit der Beine und das nichtmotorische Off zusammengefasst. In der Validierungsstudie zeigten sich noziplastische Schmerzen bei 22 % der Parkinson-Patienten [40]. Davon fielen 12 % auf die motorische Ruhelosigkeit der Beine, 6 % auf ein Dopaminagonistenentzugssyndrom und 4 % auf das nichtmotorische Off, während keine Schmerzen beim dopaminergen Dysregulationssyndrom beobachtet wurden. Bei noziplastischen Schmerzen waren, im Vergleich zu den anderen beiden Schmerzformen, eine höhere Anzahl von Körperregionen betroffen. Zudem zeigten sich Korrelationen mit den Fragebögen zum „wearing-off“, der Lebensqualität und der Ängstlichkeit.

Der vorliegende Fragebogen ermöglicht weiterhin eine Einschätzung des Schmerzes im Hinblick auf Intensität (0: kein Schmerz bis 10: schlimmster vorstellbarer Schmerz), Frequenz (1: selten, 2: manchmal, 3: häufig) und Einfluss auf den Alltag (1: leicht, 2: mäßig, 3: stark) (eAnhang 1).

## Diagnostik nicht-Parkinson-assoziierter Schmerzen

Wenn keine Assoziation der Schmerzen mit der Parkinson-Erkrankung festgestellt werden kann und trotz der Optimierung der dopaminergen Therapie weiter Schmerzen bestehen, muss an andere Schmerzformen gedacht werden. Dabei sind vor allem degenerativ bedingte Rückenschmerzen, Gelenkschmerzen bei Arthrose und die Polyneuropathie zu beachten. Durch die Fehlhaltung kann es zu spondylogenen oder radikulären Schmerzen v. a. lumbal kommen, auch wenn sich mit dem PSK-Fragebogen kein Zusammenhang mit der Parkinson-Krankheit herstellen lässt. Diagnostisch helfen klinische Untersuchung, Neurographie, Labordiagnostik und Bildgebung (z. B. lumbal und artikulär) weiter. Eine mögliche Ursache für eine Polyneuropathie kann übrigens auch ein Vitamin-B12-Mangel sein, der durch langfristige hohe L‑Dopa-Gabe verursacht werden kann [48].

## Validierungsstudie

**Figure 2:**
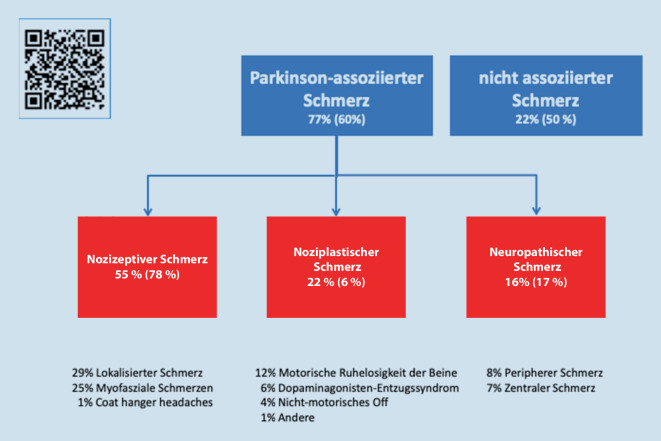

In einer multizentrischen Validierungsstudie wurde der Fragebogen zur PSK mit anderen Schmerzfragebögen (Brief Pain Inventory [BPI] und dem McGill Pain Questionnaire [MPQ]), einem Lebensqualitätsfragebogen (Parkinson’s Disease Questionnaire-8), motorischen Fragebögen (Movement Disorders Society sponsored revision of the Unified Parkinson’s Disease Rating Scale [MDS-UPDRS] III und IV und dem Wearing-off Questionnaire) und einem Depressionsfragebogen (Hospital Anxiety and Depression Scale) verglichen [40]. Intrarater- und Interraterreliabilität wurden durch eine erneute Testung durch den ersten Rater und einen weiteren Rater nach 7 Tagen bestimmt. Ausschlusskriterien waren eine Demenz (Mini-Mental State Examination < 24 Punkte), die tiefe Hirnstimulation (THS) oder Duodopa-Pumpentherapie. An der Studie nahmen 159 stationäre und ambulante Parkinson-Patienten (Krankheitsdauer 10,2 ± 7,6 Jahre) und 37 gesunde Kontrollprobanden in vier Zentren teil (drei Zentren in der Schweiz und ein Zentrum in Brasilien). 122 Patienten zeigten Parkinson-assoziierte Schmerzen (77 %), wobei 24 (15 %) Patienten mehr als ein Schmerzsyndrom angaben. Nozizeptiver, neuropathischer und noziplastischer Schmerz traten bei 87 (55 %), 25 (16 %) bzw. 35 (22 %) Patienten auf. Parkinson-unabhängige Schmerzen konnten bei 35 (22 %) Patienten festgestellt werden (Abb. 2). Der Schmerzscore korrelierte signifikant mit den Schmerzratings im BPI und im MPQ, mit motorischen Fluktuationen und Dyskinesien (MDS-UPDRS IV), der Lebensqualität, dem Depressions- und dem Ängstlichkeitsscore. Es fand sich eine moderate Inter- und Intraraterreliabilität. Gründe dafür könnten eine tageszeitlich unterschiedliche Bewertung, Wirkungsfluktuationen sowie tatsächliche Unterschiede nach einer Woche sein. Die Studie zeigte aber, dass der PSK-Fragebogen ein valides und genügend reliables Werkzeug ist, um Parkinson-assoziierten Schmerz von nicht-Parkinson-assoziiertem Schmerz zu unterscheiden und die Schmerzen einer Kategorie zuzuordnen.

### Übersetzung des Schmerzfragebogens ins Deutsche

Zwei Autoren der Originalversion (VM, CM) haben diese getrennt voneinander ins Deutsche übersetzt (s. Zusatzmaterial online). Beide Fassungen wurden anschließend verglichen und eine Konsensversion erarbeitet. Dabei wurde versucht, möglichst am Originaltext zu bleiben, damit eine inhaltliche Rückübersetzung erleichtert wird. Diese deutsche Übersetzung wurde dann bei fünf Patienten klinisch eingesetzt, um etwaige Verständnisprobleme herauszufinden. Diese wurden dann in der ersten deutschen Fassung berücksichtigt. Anschließend erfolgte von zwei weiteren Neurowissenschaftlern (CSB, FB) mit Schwerpunkt im Bereich Bewegungsstörungen eine Rückübersetzung der Version ins Englische. Auch hier wurde eine Konsensversion gebildet. Aufgrund von Diskrepanzen in der Rückübersetzung wurde die deutsche Version an einigen Stellen leicht modifiziert, um eine eindeutigere Rückübersetzung zu ermöglichen. Diese neue Form der Rückübersetzung wurde dann den Autoren der Originalpublikation (DCD, SPL) für ihr Einverständnis vorgelegt. Dabei ergab sich, dass die Frage 1 nicht eindeutig gestellt ist, wenn die Erklärungen nicht beachtet werden. Diese wurden deshalb ergänzend übersetzt (eAnhang 2). Um den Zusammenhang der Schmerzen mit dem Beginn der Erkrankung zu klären, haben wir den Ausdruck „direkt“ eingeführt. Zudem wurde ergänzt, dass drei verschiedene Schmerztypen definiert werden können (eAnhang 1).

Anschließend erfolgte die Anwendung des Fragebogens bei 30 Patienten im Alter von 71,8 ± 9,2 Jahren (70 % männlich) mit einer Krankheitsdauer von 8,9 ± 5,7 Jahren und einem durchschnittlichen Hoehn-und-Yahr-Stadium von 2,5 ± 0,8 (1–4). Bei 26 Patienten (87 %) lagen im Mittel 1,3 Schmerztypen vor (1,3 ± 0,6). Von insgesamt 35 Schmerztypen waren 18 Parkinson-assoziiert und 17 nicht-Parkinson-assoziiert. 18/30 (60 %) Patienten gaben Parkinson-assoziierte (Dauer 4,4 ± 5,7 Jahre) und 15/30 Patienten (50 %) nicht-Parkinson-assoziierte Schmerzformen an (Dauer 5,1 ± 5,7 Jahre). Sechs Patienten mit Parkinson-assoziierten Schmerztypen (20 %) gaben zusätzlich nicht-Parkinson-assoziierte Schmerztypen an (zwei davon hatten zwei nicht-Parkinson-assoziierte Schmerzformen). Ein Patient berichtete zwei Parkinson-assoziierte Schmerztypen. Bei den Parkinson-assoziierten Schmerztypen waren 14/18 (78 %) nozizeptiv, 3/18 neuropathisch (17 %) und 1/18 (6 %) noziplastisch. Die Bewertung anhand des PSK-Fragebogens lag bei den nozizeptiven Schmerzformen im Mittel bei 36, bei neuropathischen bei 33, bei noziplastischen bei 2 und bei nicht-Parkinson-assoziierten Schmerzformen bei 22 (Intensität × Frequenz × Einfluss auf den Alltag). Im Vergleich zur Validierungsstudie lässt sich der geringere Anteil noziplastischer Schmerztypen durch die etwas geringere Erkrankungsdauer und den etwas höheren Anteil ambulanter (gering betroffener), älterer und männlicher Patienten erklären [47]. An dieser Befragung nahmen, anders als in der Validierungsstudie, auch Patienten mit THS teil, die wenig über Schmerzen klagten. Zudem werden noziplastische Schmerzen (z. B. im Rahmen einer Bewegungsunruhe) meist als weniger stark empfunden und oft erst auf Nachfrage berichtet, was bei der Befragung berücksichtigt werden sollte. Erfahrungen der teilnehmenden Ärzte, die neue Definition noziplastischer Schmerzen, die kurze Testung der deutschen Version sowie die moderate Interraterreliabilität bedingen die Unterschiede wahrscheinlich mit. Der PSK-Fragebogen wird vom Arzt zusammen mit dem Patienten ausgewertet, was je nach Erfahrung und Komplexität der Schmerzen 5–10 min Minuten beansprucht.

### Vergleich mit anderen Skalen

Eine erste Schmerzklassifikation beim M. Parkinson erfolgte 1986 durch Quinn [42]. In dieser Klassifikation wurden Parkinson-assoziierte Schmerzen anhand von Off-Phasen, der Dystonie und choreatischen Dyskinesien definiert. Später entwickelte Ford eine lang verwendete Schmerzklassifikation, die muskuloskeletale Schmerzen, dystonieassoziierte Schmerzen, zentrale Schmerzen, radikuläre Schmerzen und die Akathisie unterscheidet [23, 24]. In einer neueren Einteilung wurden von Wasner und Deuschl neuropathische, nozizeptive und verschiedene Schmerzen unterschieden [52]. Zuletzt wurde auch die in unserer Studie verwendete Einteilung vorgeschlagen (neuropathisch, nozizeptiv, noziplastisch), wobei die Autoren noch zwischen Parkinson-spezifischen und nicht-Parkinson-spezifischen Schmerzen unterschieden haben [34].

Im Jahr 2015 wurde mit dem King’s Pain Questionnaire ein erster Schmerzfragebogen für Schmerzen bei Patienten mit M. Parkinson validiert [13]. In diesem Fragebogen werden sieben verschiedene Schmerzformen unterschieden und eine Schmerzbewertung durchgeführt. Dieser Fragebogen geht von Parkinson-assoziierten Schmerzen aus, welche anhand des Non-Motor Symptom Questionnaire (NMS-Quest) definiert wurden (Ausschluss anderer Ursachen; [12]). Im Vergleich dazu ermöglicht die PSK eine Zuordnung zu Parkinson-assoziiertem Schmerz auch anhand weiterer Faktoren [16, 37]. Eine Unterteilung von Parkinson-assoziiertem Schmerz nach der zugrunde liegenden Schmerzkategorie ist hiermit erstmals in einem Fragebogen möglich. Diese kann dann weitere diagnostische und therapeutische Maßnahmen vereinfachen.

## Therapie Parkinson-assoziierter Schmerzen

**Figure 3:**
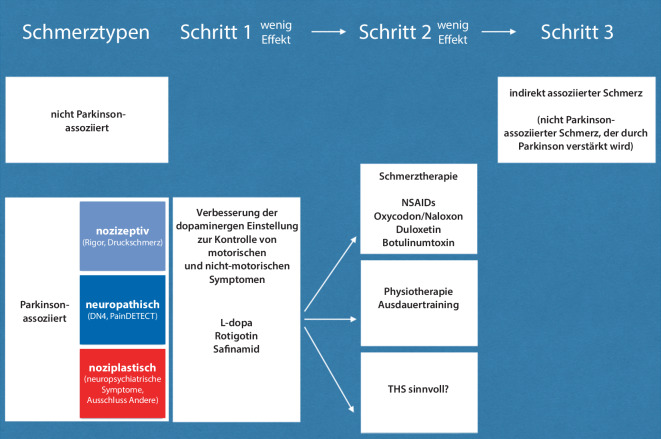

In der Therapie von Schmerzen bei M. Parkinson können verschiedene Therapieansätze je nach Wirksamkeit und Therapieadhärenz kombiniert werden ([39]; Abb. 3). Im Wesentlichen bestehen die Therapien aus medikamentöser Therapie, neurorehabilitativen Maßnahmen und aus der Anwendung apparategestützter Verfahren [8, 28].

Wenn eine Assoziation der Schmerzen insbesondere mit einer Off-Phase oder ein Ansprechen der Schmerzen auf die dopaminerge Medikation gezeigt werden kann (meist nozizeptive oder noziplastische Schmerzen), dann ist die bessere *Einstellung der motorischen und nichtmotorischen Symptome* zielführend. Insbesondere sind hier Umstellungen der Medikation geeignet, die die Off-Phasen reduzieren. Gelegentlich kann auch bei guter Einstellung der Motorik durch eine weitere Erhöhung der dopaminergen Medikation ein Effekt auf die Schmerzen beobachtet werden. Auch ist der Einsatz bestimmter dopaminerger und anderer Medikamente geeignet, wie zuletzt in umfassenden Reviews gezeigt werden konnte [8, 43]. Entsprechende Studien konnten hier einen signifikanten Effekt vor allem für Rotigotin zeigen [30, 51]. Für Pramipexol lagen die nichtsignifikanten Effekte in einer ähnlichen Größenordnung [2]. Ebenfalls wurden für Safinamid schmerzreduzierende Effekte beschrieben [10].

Bei Dystonien kann auch Botulinumtoxin eine Möglichkeit sein. Bei neuropathischen Schmerzen können entsprechende Medikamente zum Einsatz kommen (Pregabalin, Gabapentin), die aber noch nicht in Studien untersucht wurden [9]. Bisher hat sich erst Duloxetin bei zentralem neuropathischem Schmerz in einer Open-label-Studie als wirksam erwiesen [21]. Eine mögliche Zweitlinienalternative für Schmerzen bei M. Parkinson kann die Fix-Kombination von Oxycodon mit Naloxon sein, wobei Obstipation und Delirien als mögliche Nebenwirkungen bedacht werden müssen [50].

Parallel zur besseren Einstellung der Parkinson-Medikation wird *regelmäßiges körperliches Training* empfohlen, denn Studien konnten erstaunliche Effekte des Trainings auf Schmerzen bei der Parkinson-Krankheit zeigen. Ursache dieses Effektes scheint die anstrengungsinduzierte Schmerzunempfindlichkeit („exercise-induced hypoalgesia“) zu sein, die zuletzt schon durch kurzzeitiges Training auch für den M. Parkinson gezeigt werden konnte [41]. In der wichtigsten der bisher vorliegenden Therapiestudien bei Patienten im Hoehn-und-Yahr-Stadium 2 bis 3 wurden drei verschiedene Therapieprogramme an drei Tagen (à 70 min)/Woche über 6 Monate verglichen [44]. Je 30 Patienten nahmen entweder an einem Laufprogramm, am Nordic Walking oder an einem Flexibilitäts- und Entspannungsprogramm teil. 70 % der Patienten gaben Schmerzen zu Beginn der Intervention an, wobei alle Interventionen einen guten schmerzreduzierenden Effekt für Nackenschmerzen, Hüft- und iliosakrale Schmerzen hatten. Der schmerzreduzierende Effekt war besonders ausgeprägt in der Gruppe der Nordic-Walking-Patienten, von den 30 % nach der Intervention keinen Schmerz mehr angaben. Auch konnten das Laufprogramm und das Nordic Walking stärkere Effekte auf Rücken‑, Hand- und Beinschmerzen als das Flexibilitäts- und Entspannungsprogramm bewirken. Interessanterweise hat sich die *THS*, auch unabhängig vom Effekt auf motorische Funktionen, als sehr wirksam in der Langzeitbehandlung Parkinson-assoziierter Schmerzen gezeigt [15, 17]. Funktionelle MRI-Studien weisen darauf hin, dass die Wirkweise auf einer stimulationsinduzierten Hemmung der primär somatosensorischen Aktivität beruht [20].

## Schlussfolgerung

Die neue Parkinson-Schmerzklassifikation ermöglicht erstmalig anhand eines hierarchisch aufgebauten Fragebogens (1. Parkinson-Assoziation, 2. Schmerzmechanismus und 3. Schmerzintensität) eine reliable und valide Zuordnung, welche diagnostisch und therapeutisch relevant ist [40]. Der Fragebogen ist kurz und einfach zu handhaben, was eine breite Anwendung in der Praxis ermöglichen soll. Eine Kurzversion des PSK-Fragebogens liegt auch online vor (siehe QR-Code in Abb. 2). Parkinson-assoziierte Schmerzen haben meist einen nozizeptiven Charakter und sollten primär durch die Anpassung der dopaminergen Medikation behandelt werden, bevor andere medikamentöse Therapien zum Einsatz kommen [39]. Insbesondere die THS und Ausdauersport haben einen sehr guten Effekt zeigen können. Zudem sind Erkennung und Behandlung nicht-Parkinson-assoziierter Schmerzen wichtig.

## Supplementary Information





