# Supplementary material for: The new Parkinson’s disease pain classification system (PD-PCS)
Source: Nervenarzt. 2022 Jan 28;93(10):1019–27. [Article in German] doi: 10.1007/s00115-021-01258-y (PMC9534980; doi:10.1007/s00115-021-01258-y)
Supplement: Supplementary file 1 [file 115_2021_1258_MOESM1_ESM.docx]

**Anhang 1. Parkinson-Schmerzklassifikations-Fragebogen**

**Parkinson-Schmerzklassifikations-Fragebogen**

Name Geburtsdatum Datum

Krankheitsdauer Seitenbetonung

Parkinson-Medikation

Ihr Patient hat chronische Schmerzen. Bitte machen Sie separate Angaben für jeden Schmerztyp und berücksichtigen Sie Krankheitsdauer, Seitenbetonung und Behandlung. Geben Sie unten die Schmerzdauer und -lokalisation für jeden Typ an. Nachfolgend können drei verschiedene

Schmerztypen angegeben werden.

|  | Schmerz 1 | Schmerz 2 | Schmerz 3 |
| --- | --- | --- | --- |
| Lokalisation |  |  |  |
| Bestehend seit |  |  |  |

**Schritt 1:** Anhand von vier Fragen soll der Zusammenhang mit der Parkinson-Erkrankung festgestellt werden (mindestens eine Frage muss mit “ja” beantwortet werden, um zu **Schritt 2** überzugehen; ansonsten steht der Schmerz nicht im Zusammenhang mit der Parkinson-Erkrankung – **Schritt 3**).

|  | Schmerz 1 | Schmerz 2 | Schmerz 3 |
| --- | --- | --- | --- |
| Hat Ihr Schmerz (direkt) nach dem Auftreten der Parkinson-Symptome begonnen oder hat er sich dadurch verstärkt? |  |  |  |
| Ist Ihr Schmerz stärker, wenn Steifheit (Rigor), Zittern (Tremor) oder die Verlangsamung Ihrer Bewegungen ausgeprägter sind? |  |  |  |
| Steht Ihr Schmerz mit abnormalen Bewegungen (sog. Dyskinesien) im Zusammenhang? |  |  |  |
| Verbessern sich Ihre Schmerzen nach der Einnahme von Parkinson-Medikamenten? |  |  |  |

**Schritt 2:** Bitte klassifizieren Sie den Parkinson-assoziierten Schmerz anhand der Mechanismen

**Neuropathischer Schmerz (DN4 ≥ 4 Punkte)**

|  | Schmerz 1 | Schmerz 2 | Schmerz 3 |
| --- | --- | --- | --- |
| Zentral |  |  |  |
| Peripher |  |  |  |
| Score:  Intensität x Frequenz x Auswirkung |  |  |  |

**Nozizeptiver Schmerz (Druckschmerzhaftigkeit)**

|  | Schmerz 1 | Schmerz 2 | Schmerz 3 |
| --- | --- | --- | --- |
| Lokalisierter Schmerz |  |  |  |
| Myofasziale Schmerzen |  |  |  |
| Coat hanger headaches |  |  |  |
| Score:  Intensität x Frequenz x Auswirkung |  |  |  |

**Noziplastischer Schmerz (Ausschluss anderer Mechanismen und neuropsychiatrische Symptome)**

|  | Schmerz 1 | Schmerz 2 | Schmerz 3 |
| --- | --- | --- | --- |
| Nicht-motorisches Off |  |  |  |
| Motorische Ruhelosigkeit der Beine |  |  |  |
| Dopaminagonisten-Entzugssyndrom |  |  |  |
| Anderes |  |  |  |
| Score:  Intensität x Frequenz x Auswirkung |  |  |  |

**Schritt 3:** **Nicht Parkinson-assoziierter Schmerz**

|  | Schmerz 1 | Schmerz 2 | Schmerz 3 |
| --- | --- | --- | --- |
| Lokalisation |  |  |  |
| Mechanismus |  |  |  |
| Score:  Intensität x Frequenz x Auswirkung |  |  |  |

**Neuropathischer Schmerz** wird durch eine Läsion oder Krankheit des somatosensorischen Nervensystems verursacht. Neuropathischer Schmerz wird mittels eines positiven Ergebnisses im Neuropathischen Schmerz-Fragebogen DN4 diagnostiziert (DN4 ≥ 4 Punkte). Zentraler und peripherer neuropathischer Schmerz kann anhand der Lokalisation (peripherer Nerv, Nervenwurzel, distal-symmetrisch vs. diffuse zentrale Verteilung) unterschieden werden.

**Neuropathischer Schmerz-Fragebogen** (Douleur Neuropathique-4 questionnaire (DN-4)): Kann man die Schmerzen wie folgt beschreiben? 1. Brennend; 2. Wie schmerzhafte Kälte; 3. Wie elektrische Schläge. Treten die folgenden Beschwerden zusammen mit den Schmerzen im selben Körperbereich auf? 4. Kribbeln (Ameisenlaufen); 5. Pieksen (wie tausend Nadelstiche); 6. Taubheitsgefühl; 7. Juckreiz. Sind die Schmerzen in einem Bereich lokalisiert, in dem die körperliche Untersuchung Folgendes zeigt? 8. Hypoästhesie bei Berührung; 9. Hypoästhesie bei Nadelreizen (pieksen). Werden die Schmerzen ausgelöst oder verschlimmert durch? 10. Bestreichen der Haut mit einem Pinsel.

**Nozizeptiver Schmerz** entsteht durch eine tatsächliche oder drohende Verletzung nicht-neuralen Gewebes mittels der Aktivierung von Nozizeptoren. Hat der Patient Schmerzen bei der Palpation von Muskeln, Sehnen, Faszien oder eine schmerzhafte Steifheit? Dieses beinhaltet muskuloskleletale Schmerzen aufgrund von Wirkungsfluktuationen wie Schmerzen im Off-Zustand (morgendliche Akinesie, Schmerzen während des wearing-off) und unterschiedliche schmerzhafte Dystonien (morgendliche Dystonie, andere Off-Dystonie, Dystonie kurz vor oder nach der Medikamenteneinnahme) sowie Schmerzen bei Überbewegungen (peak-dose pain). Lokalisierte und regionale Schmerzsyndrome, myofasziale Schmerzen und der Coat hanger headache (bei orthostatischer Hypotonie) gehören ebenfalls zu den nozizeptiven Schmerzen.

**Noziplastischer Schmerz** wird diagnostiziert, wenn weder neuropathischer noch nozizeptiver Schmerz vorliegen. Im klinischen Alltag sind diese Schmerzen Folge von hyper- oder hypodopaminergen Fluktuationen, wenn nicht-motorische neuropsychiatrische Symptome dominieren und der Schmerz nicht einziges Symptom, sondern Teil einer komplexen klinischen Symptomatik ist. Der Patient kann Schweißausbrüche, Dysphorie, innere Unruhe, motorische Ruhelosigkeit und einen tief im Abdomen oder im Gesicht gelegenen, schlecht lokalisierbaren oder schnell wandernden Schmerz aufweisen. In den meisten Fällen liegen ein Dopaminagonisten-Entzugssyndrom, ein Dopamin-Dysregulationssyndrom oder andere neurosychiatrische Auffälligkeiten vor. Wir klassifizieren die motorische Ruhelosigkeit der Beine und das nicht-motorische Off auch hier, wenn die neuropathische Schmerzkomponente nicht im Vordergrund steht.

**Bewertung der Schmerztypen**

Bewerten sie jeden Schmerztyp gemäß Intensität (0: 'kein Schmerz' und 10: 'schlimmster vorstellbarer Schmerz', wählen Sie die Intensität von Ihrem Schmerz), Frequenz (1: selten, 2: manchmal, 3: häufig) und Auswirkung auf Ihr Alltagsleben (1: leicht, 2: mässig, 3: stark). Der Gesamt-Punktwert für jeden Schmerztyp ergibt sich durch die Multiplikation von Intensität, Frequenz und Auswirkung mit einer Spannbreite von 0-90 Punkten.

Nach Mylius V, Perez-Lloret S et al. Pain 2021
