# Supplementary material for: The new Parkinson’s disease pain classification system (PD-PCS)
Source: Nervenarzt. 2022 Jan 28;93(10):1019–27. [Article in German] doi: 10.1007/s00115-021-01258-y (PMC9534980; doi:10.1007/s00115-021-01258-y)
Supplement: Supplementary file 2 [file 115_2021_1258_MOESM2_ESM.docx]

**Anhang 2. Erläuterung zur Parkinson-Schmerz-Klassifikation**

**Schritt 1:** **Anhand von vier Fragen soll der Zusammenhang mit der Parkinson-Erkrankung festgestellt werden.**

1. Hat Ihr Schmerz (direkt) nach dem Auftreten der Parkinson-Symptome begonnen oder hat er sich dadurch verstärkt? Der enge zeitliche Zusammenhang zwischen Schmerzbeginn und Erkrankungsbeginn kann Hinweise auf erste motorische Symptome des M. Parkinson geben [[5](#_ENREF_5)].
2. Ist Ihr Schmerz stärker, wenn Steifheit (Rigor), Zittern (Tremor) oder die Verlangsamung Ihrer Bewegungen ausgeprägter sind? Im Off (niedriger Dopaminspiegel) kann der Schmerz während des wearing-off, der end-of-dose Akinesie (morgendliche oder nächtliche Akinesie oder in Abhängigkeit von der Medikamenteneinnahme), des paroxysmalen Off (unabhängig von der Medikamenteneinnahme) oder während einer Off-Dystonie (oft am frühen Morgen) auftreten [[3](#_ENREF_3), [6](#_ENREF_6), [9](#_ENREF_9), [13](#_ENREF_13), [19](#_ENREF_19)].
3. Steht Ihr Schmerz mit abnormalen Bewegungen (sog. Dyskinesien) im Zusammenhang? Im On (hohe dopaminerge Stimulation) kann es zu choreatischen Dyskinesien kommen, die normalerweise nicht als schmerzhaft empfunden werden. Wenn jedoch beispielsweise eine Arthrose vorliegt, können diese auch Schmerzen verursachen. Choreatische Dyskinesien beeinhalten peak-dose, Plateau- und biphasische Dyskinesien, wobei letztere bei intermediären Dopaminspiegeln auftreten. Eine On-Dyskinesie, besonders die biphasische Dyskinesie, kann sich auch als schmerzhafte Dystonie äussern. Nur selten kommen choreatische und dystonische Dyskinesien gleichzeitig vor [[9](#_ENREF_9), [13](#_ENREF_13)].
4. Verbessern sich Ihre Schmerzen nach der Einnahme von Parkinson-Medikamenten? Die Fluktuation der Schmerzen mit der dopaminergen Stimulation kann auch ein Hinweis für eine Assoziation zwischen Schmerzen und der Parkinson-Erkrankung sein. Hierbei soll jeder positive Effekt der medikamentösen Parkinsonbehandlung auf die Schmerzen einfliessen [[2](#_ENREF_2), [19](#_ENREF_19)].

**Schritt 2: Bitte klassifizieren den Parkinson-assoziierten Schmerz gemäss den folgenden drei Mechanismen**

1. **Neuropathischer Schmerz** wird durch eine Läsion oder Krankheit des somatosensorischen Nervensystems verursacht [[16](#_ENREF_16)]. Neuropathischer Schmerz wird mittels eines positiven Ergebnis im Neuropathischen Schmerz-Fragebogen DN4 diagnostiziert (DN4 ≥ 4), unabhängig von einer bestehenden schmerzhaften Palpation [[1](#_ENREF_1)]. Zentraler und peripherer neuropathischer Schmerz können anhand ihrer Lokalisation (peripherer Nerv, Nervenwurzel, distal-symmetrisch vs. diffuse zentrale Verteilung) unterschieden werden.
2. **Nozizeptiver Schmerz** entsteht durch eine tatsächliche oder drohende Verletzung nicht-neuralen Gewebes durch die Aktivierung von Nozizeptoren [[8](#_ENREF_8)]. Hat der Patient Schmerzen bei der Palpation von Muskeln, Sehnen, Faszien oder eine schmerzhafte Steifheit? Dieses beinhaltet muskuloskeletale Schmerzen aufgrund von Wirkungsfluktuationen wie z.B. Schmerzen im Off-Zustand (morgendliche Akinesie, Schmerzen während des wearing-off), unterschiedliche schmerzhafte Dystonien (morgendliche Dystonie, andere Off-Dystonie, Dystonie kurz vor oder nach der Medikamenteneinnahme) sowie Schmerzen bei Überbewegungen (peak-dose pain) [[13](#_ENREF_13)]. Lokalisierte und regionale Schmerzsyndrome, myofasziale Schmerzen und der Coat hanger headache (bei Hypotonie) gehören ebenfalls zu den nozizeptiven Schmerzen. Wenn Gelenk-, Faszien-, Sehnen- oder Muskelpalpation schmerzhaft ist, wird nozizeptive Information durch die Aktivierung von Nozizeptoren weitergeleitet (C- oder A-delta Fasern der Haut oder tieferer Gewebe). Häufig liegt dabei eine sekundäre mechanische Hyperalgesie um die schmerzhafte Region vor. Ursächlich wird eine zentrale Sensibilisierung vermutet, die von der dopaminergen Stimulation abhängig ist [[17](#_ENREF_17)]. Diese verändert die Schmerzwahrnehmung durch Beeinflussung sensorischer Schmerzschwellen [[4](#_ENREF_4), [11](#_ENREF_11)].
3. **Noziplastischer Schmerz*** entsteht durch eine veränderte Schmerzweiterleitung ohne Hinweise für eine Gewebsschädigung mit Rezeptoraktivierung oder eine Erkrankung oder Läsion des somatosensorischen Systems [[10](#_ENREF_10)]. Dabei liegt eine Überaktivierung des schmerzverarbeitenden Systems, welche durch ein Ungleichgewicht von schmerz- und stimmungsverarbeitenden deszendierenden Systemen verursacht wird, vor. In der von der IASP vorgeschlagenen hierarchischen Definition, die auch in der PSK verwendet wird, kann noziplastischer Schmerz festgestellt werden, wenn der Schmerz weder neuropathisch (DN4 < 4) noch nozizeptiv ist [[8](#_ENREF_8)]. Noziplastische Schmerzen sind wahrscheinlich die Folge von hyper-/hypodopaminergen Fluktuationen, wenn nicht-motorische neuropsychiatrische Symptome dominieren und der Schmerz nicht einziges Symptom, sondern Teil einer komplexen klinischen Symptomatik ist. Der Patient kann Schweissausbrüche, Dysphorie, innere Unruhe, motorische Ruhelosigkeit und z.B. einen tief im Abdomen oder im Gesicht gelegenen, schlecht lokalisierbaren oder schnell wandernden Schmerz aufweisen. Wir vermuten, dass Patienten mit einem Dopaminagonisten-Entzugssyndrom [[12](#_ENREF_12)], einem Dopamin-Dysregulationssyndrom [[18](#_ENREF_18)] oder anderen neuropsychiatrischen Auffälligkeiten des Dopaminspiegels, wenn der Schmerz nur Teil des komplexen klinischen Bildes ist, einen noziplastischen Schmerz haben können [[15](#_ENREF_15)]. Es wird zudem angenommen, dass die motorische Ruhelosigkeit der Beine und das nicht-motorische Off zu den noziplastischen Schmerzen gezählt werden können, wenn die neuropathische Schmerzkomponente nicht im Vordergrund steht [[7](#_ENREF_7), [14](#_ENREF_14)] ******.

*Der Absatz wurde im Vergleich zur Validierungsstudie überarbeitet. Da bisher, ausser der Validierungsstudie, noch keine Studien zu noziplastischen Schmerzen beim M. Parkinson vorliegen haben wir eine vorsichtigere Formulierung gewählt.

**Die Ergebnisse der Validierungsstudie zeigen, dass die motorische Ruhelosigkeit der Beine, das nicht-motorische Off und das Dopaminagonisten-Entzugssyndrom zu den noziplastischen Schmerzen gezählt werden können.

**Literatur**

1. Bouhassira D, Attal N, Alchaar H et al. (2005) Comparison of pain syndromes associated with nervous or somatic lesions and development of a new neuropathic pain diagnostic questionnaire (DN4). Pain 114:29-36

2. Castrioto A, Thobois S, Carnicella S et al. (2016) Emotional manifestations of PD: Neurobiological basis. Movement disorders 31:1103-1113

3. Chaudhuri KR, Martinez-Martin P, Brown RG et al. (2007) The metric properties of a novel non-motor symptoms scale for Parkinson's disease: Results from an international pilot study. Movement disorders 22:1901-1911

4. Cury RG, Galhardoni R, Teixeira MJ et al. (2016) Subthalamic deep brain stimulation modulates conscious perception of sensory function in Parkinson's disease. Pain 157:2758-2765

5. Defazio G, Berardelli A, Fabbrini G et al. (2008) Pain as a nonmotor symptom of Parkinson disease: evidence from a case-control study. Arch Neurol 65:1191-1194

6. Ford B (2010) Pain in Parkinson's disease. Movement disorders 25 Suppl 1:S98-103

7. Gjerstad MD, Tysnes OB, Larsen JP (2011) Increased risk of leg motor restlessness but not RLS in early Parkinson disease. Neurology 77:1941-1946

8. IASP (2021) [https://www.iasp-pain.org/resources/terminology/ - nociplastic-pain](https://www.iasp-pain.org/resources/terminology/#nociplastic-pain).

9. Juri C, Rodriguez-Oroz MC, Burguera JA et al. (2010) Pain and dyskinesia in Parkinson's disease. Movement disorders 25:130-132

10. Kosek E, Cohen M, Baron R et al. (2016) Do we need a third mechanistic descriptor for chronic pain states? Pain 157:1382-1386

11. Mylius V, Brebbermann J, Dohmann H et al. (2011) Pain sensitivity and clinical progression in Parkinson's disease. Movement disorders 26:2220-2225

12. Pondal M, Marras C, Miyasaki J et al. (2013) Clinical features of dopamine agonist withdrawal syndrome in a movement disorders clinic. J Neurol Neurosurg Psychiatry 84:130-135

13. Quinn NP, Koller WC, Lang AE et al. (1986) Painful Parkinson's disease. Lancet 1:1366-1369

14. Rana AQ, Depradine J (2011) Abdominal pain: a symptom of levodopa end of dose wearing off in Parkinson's disease. West Indian Med J 60:223-224

15. Storch A, Schneider CB, Wolz M et al. (2013) Nonmotor fluctuations in Parkinson disease: severity and correlation with motor complications. Neurology 80:800-809

16. Treede RD, Jensen TS, Campbell JN et al. (2008) Neuropathic pain: redefinition and a grading system for clinical and research purposes. Neurology 70:1630-1635

17. Treede RD, Meyer RA, Raja SN et al. (1992) Peripheral and central mechanisms of cutaneous hyperalgesia. Prog Neurobiol 38:397-421

18. Warren N, O'gorman C, Lehn A et al. (2017) Dopamine dysregulation syndrome in Parkinson's disease: a systematic review of published cases. J Neurol Neurosurg Psychiatry 88:1060-1064

19. Wasner G, Deuschl G (2012) Pains in Parkinson disease-many syndromes under one umbrella. Nat Rev Neurol 8:284-294
